# Neuromotor adaptations in people with Parkinson's Disease following a 12-month multimodal non-contact boxing intervention

**DOI:** 10.3389/fnhum.2025.1707832

**Published:** 2026-01-21

**Authors:** Adam C. King, Joshua C. Carr, Caleb Voskuil, Kuanting Chen, Ryan R. Porter, Zoe Thijs, Christopher R. Watts

**Affiliations:** 1 Department of Kinesiology, Texas Christian University, Fort Worth, TX, United States; 2 Department of Kinesiology, Kansas State University, Manhattan, KS, United States; 3 Department of Exercise Science, Lakeland University, Plymouth, WI, United States; 4 Department of Communication Disorders and Occupational Therapy, University of Arkansas, Fayetteville, AR, United States; 5 Davies School of Communication Sciences and Disorders, Texas Christian University, Fort Worth, TX, United States

**Keywords:** Parkinson's Disease, motor function, complexity and disease, mobility, balance, force control

## Abstract

Parkinson's Disease (PD) is a common neurological disorder that diminishes neuromotor control. Exercise training provides a nonpharmacological treatment option that may help motor symptom severity and trajectory. In the present case series, we assessed linear and non-linear measures of neuromotor control along with functional measures of mobility in six men living with PD across a year of multimodal exercise training. Our measurements encompassed mobility, balance, strength, and force control metrics and were captured at baseline as well as at defined intervals following exercise engagement. The results appear to indicate a favorable preservation of functional ability and neuromotor control for most of the cohort across the yearlong exercise intervention. Our linear measures of neuromotor control generally remained stable with slight improvements from baseline to month 12, with visually distinct trends for our non-linear measures (Sample Entropy) of neuromotor control suggestive of more adaptive motor control strategies for both postural and force control. These data corroborate reports showing favorable outcomes for people with Parkinson's Disease following exercise engagement. Our year-long assessment of neuromotor control in a cohort of men with Parkinson's disease provides a novel contribution to the trajectory of change in this population while undergoing multimodal exercise training.

## 1 Introduction

Parkinson's Disease (PD) is the second most common neurological disorder worldwide and is often characterized by four cardinal motor symptoms—tremor, rigidity, bradykinesia, and posture and gait disturbances. Cascading impacts of these symptoms alter a myriad of musculoskeletal function abilities, including impaired walking (Allen et al., 2010), diminished postural stability (Dibble et al., 2009), and reduced maximal strength (Helgerud et al., 2020), contributing to fall-related injuries and limited engagement in physical activity (Crizzle and Newhouse, 2006). However, as a complementary therapy and adjunctive treatment to pharmacological approaches, exercise appears to attenuate the natural neurological declines of PD progression. To date, while diverse exercise programs have been explored (Rafferty et al., 2017; Ellis et al., 2021), a clear consensus is lacking on the recommended dosage (type, duration, intensity, etc.) of exercise involvement for People with PD (PwPD). Further exploration around novel interventions that impact overall neuromotor function is needed.

PD is a progressive neurodegenerative disease that displays impaired neural function including, but not limited to, reduced force production capabilities with underlying mechanisms linked to alternations in voluntary muscle activation (Folland et al., 2011), increased antagonist muscle coactivation (Rose et al., 2013), and degraded motor unit discharge behavior (Folland et al., 2011). Such neural changes significantly impact performance of functional activities (Alves et al., 2005; Velseboer et al., 2013). Impaired sensorimotor control in PD leads to difficulties with daily living activities (i.e., buttoning, eating, extracting money from a wallet, handling keys, cooking) as well as with gross motor tasks (standing balance and gait). Yet, the mechanisms underlying these neural adaptations remain elusive and require investigation of metrics beyond those typically used for clinical evaluation. While positive neural alterations with exercise interventions occur for PwPD, examining the impact of long-term therapeutic exercise programs on attenuating the progressive loss of neural control may better inform intervention options for individuals living with PD.

Physical exercise is a strongly supported evidence-based intervention option that is becoming a standard recommendation for disease management. Specifically, community-based activities, such as karate (Fleisher et al., 2020), tai chi (Li et al., 2012), aquatic classes (Carroll et al., 2017), and dancing (Shanahan et al., 2017) represent exercise programs that have the potential to target neuroplastic modifications in PwPD as well as enhance numerous quality-of-life indicators (Hermanns et al., 2021; Larson et al., 2022). Additionally, the social community and accountability of group exercise classes foster long-term exercise adherence, especially when participants are grouped with individuals of similar age (Farrance et al., 2016; Beauchamp et al., 2018). Recent developments with multi-modal, non-contact boxing programs encapsulate these aspects (Combs et al., 2013; Stegemöller et al., 2017; Domingos et al., 2019) as reflected by the multimodal exercise regime that includes aspects of high-intensity exercise, flexibility and stretching, speed, balance and footwork, strength training, and endurance. Furthermore, multi-modal, non-contact boxing targets numerous aspects of functional, independent living and fosters cognitive stimulation, especially compared to other exercise approaches, like treadmill walking or stationary biking (Domingos et al., 2019).

To better understand contributing factors that attenuate PD progression, it is important to examine the underlying mechanisms associated with the longitudinal adaptations when individuals engage in prolonged exercise interventions. To address this gap, we evaluated neuromotor function as a result of participation in a multimodal, non-contact boxing exercise program. Components of neuromotor function evaluation included aspects of clinical evaluation (reach/step performance, mobility—walking speed and sit-to-stand, and muscular strength) and laboratory assessments (postural control and force steadiness) to understand functional activities as well as the overall state of the nervous system for PwPD. Based on the exercise activities of the multi-modal boxing program, we hypothesized that at minimum exercise engagement would attenuate the normal functional declines of PD progression and positively alter neuromotor function.

## 2 Materials and method

### 2.1 Design of the study

This study used a case series design to examine neuromotor control across 12 months of multimodal exercise training in males living with PD. These data were collected as part of a multidisciplinary collaboration with independent measures and hypotheses, the results of which has been published elsewhere (Watts et al., 2023). Our design used a double baseline assessment following a familiarization session all of which were completed over the course of 2 weeks. Following the baseline assessments, participants were assessed after 1-, 2-, 3-, 6-, 9-, and 12-months following exercise training commencement. Each testing session consisted of the same measurements, with consideration given for time of day, medication status, and general testing readiness. Additionally, participants were screened with the Mini-Mental State examination (MMSE) and achieved scores of 25 or greater. Each participant verified their ability to attend two exercise classes per week, which were at no cost to the study participants. The study was approved by the Institutional Review Board for Human Subjects Research at Texas Christian University. All participants read and signed an Informed Consent document that including confirmation of voluntary involvement and consent to publish obtained data.

### 2.2 Participants

Eight adult males diagnosed with Parkinson's disease participated in this study with one subject withdrawing due to a non-related back injury, while another participant was excluded from the dataset due to compliance issues (>1 month of exercise training missed). Inclusion criteria for all participants were: diagnosed with Parkinson's Disease by a neurologist, not currently involved in an exercise program, were currently undergoing dopamine-replacement therapy, and able attend the exercise program twice per week for the duration of the intervention. At the beginning of the study, none of the participants had any neurological diagnoses other than Parkinson's disease, and none had engaged in a structured exercise program for the past 6 months. None of the participants were receiving physical therapy, all were able to walk independently, and they were all residing in their respective communities.

### 2.3 Multimodal exercise training

The multimodal exercise training was based on the tenets of Punching out Parkinson's (Salvatore et al., 2022). Each training session spanned 60 min and was divided into seven stations of roughly equal duration. Stations collectively incorporated flexibility work, aerobic activity, and resistance exercises. Participants systematically cycled through the stations consisting of a dynamic warm-up with stretching, footwork drills, heavy-bag work, trainer-led hand-mitt exercises, the speed bag, a resistance-training segment, and a final cool-down period. Sessions were completed in small groups, and exercise modifications were provided individually for participants of differing ability and fitness while progressing in difficulty throughout the sessions. Participants attended two sessions per week over a 12-month period, accumulating approximately 120 min of activity weekly, 480 min monthly, and about 5,750 min across the intervention.

### 2.4 Outcome measurements

Our measures of neuromotor control are derived from submaximal isometric force control of the hands and postural control during standing. The order of testing was always the same across the study protocol and was structured to assess postural control first and then force control of the hands.

The 6-meter walking test (6MWT) was performed with instructions stating to walk at a safe, comfortable pace as quickly as possible. A verbal command prompted walk initiation and the participant walked toward a cone located 6 m from the starting position while two researchers recorded the time to completion.

The five times sit-to-stand (FTSTS) task is an objective measure to determine when an individual with PD may be at risk of falling and provides a correlate for lower extremity strength. Participants began the FTSTS test in a seated position on an armless chair with arms folded across their chest and feet firmly placed on the ground at about hip width apart. Individuals were instructed to move into a full-standing position with hips and knees in full extension, then return to the seated position and repeat the process five times as quickly as possible without using the upper limbs. Two researchers recorded the total time for FTSTS, and the average time was computed for performance outcome.

The Multidirectional Reach Task (MDRT) is a valid assessment of stability (Newton, 2001) whereby individuals need to voluntarily reach in four different directions with the feet stationary, resulting in changes to the center-of-gravity to the limits of the stability boundary. Instructions include statements such as: “without moving your feet or taking a step, reach as far as you can and try to keep your hand along the yardstick.” For the backward direction, the subject is asked to “lean as far back as you can.” A meter stick placed at shoulder height on the wall was used to measure starting position and maximal reach distance for all four (forward, backward, right lean, and left lean) directions. The researcher recorded performance for all visits.

The Maximum Step Length (MSL) task assesses stepping ability and serves as an indicator of mobility function and fall risk in older adults. Participants performed the MSL test in the forward, lateral, and backward directions for stepping distance. Five trials were performed for each direction with the maximal step distance used as the MSL score.

Standing balance assessment was completed using a force plate form (OR6-7, AMTI, Boston, MA) with a sampling frequency set at 100 Hz. Balance tests were conducted in the following conditions: (a) two-legged upright standing with eyes open (EO), (b) two-legged upright standing with eyes closed (EC), and (c) two-legged upright standing with eyes open on a foam pad (FM). Each balance task was performed for two trials of 20s duration with rest intervals (~30s) were provided during trials. Center of pressure (COP) data were exported to MATLAB () for processing through custom written programs that filtered (10 Hz low-pass Butterworth filter) and conducted the linear (e.g., AREA) and non-linear analyses (e.g., sample entropy) that index postural control.

To assess force control, participants performed isometric index finger abduction in a custom-built testing apparatus while in a seated position. For this assessment, the strength of both index fingers was measured with miniature compression loadcells (Model LBM; Interface, AZ) while the palms of the hands were placed on the table with the index finger producing abductive forces. The order of testing between hands was randomized for each visit. After determining index finger abduction strength, the participants performed a series of submaximal contractions at 30% of the maximal strength value for each finger while the participants matched their force output on a trapezoidal force template displayed on a TV screen directly in front of them. These tracings were non-fatiguing and were performed approximately three times with each finger. The participants were asked to match their force tracing as closely as possible to the visual template, with specific instructions to be as steady as possible. The trapezoid tracing required a three second rise, an eight second plateau hold, and a three second descent. Force control was quantified as the steadiest three-second epoch of the plateau phase with custom scripts (MATLAB 2023a, Mathworks). The outcome measures from this assessment are the coefficient of variation (CoV%), and Sample Entropy (SE).

Characterizing the nature of neuromotor complexity within the COP and force control trajectories provides valuable information to our understanding of health and function (Stergiou et al., 2006). In contrast to standard magnitude-based metrics, sample entropy indexes the structural dynamics of control that consists of sensory feedback and feedforward control mechanisms. Previous work using non-linear analyses have shown sensitivity to detect subtle motor impairments between various population groups, including PD (Schmit et al., 2006; Bartsch et al., 2007).

### 2.5 Statistical analyses

Within our case series, we report effect sizes (shown as Cohen's *d*) for each intervention time point in comparison to the respective baseline value and were computed for our measures of functional performance, postural sway, and isometric force control across the year. Descriptive statistics of means ±95% confidence intervals (CI) are shown in the figures to highlight the observed trends throughout the boxing intervention. Using CI in the descriptive statistics aligns with the small sample size of our data set and offers a more accurate representation of the range of values compared to reporting standard deviations. While yielding greater uncertainty, CI does offer the benefits of providing a transparent view of the precision of the findings along with the effect size magnitude. Cohen's *d v*alues are reported to demonstrate effect-size, and the threshold values were trivial (< 0.2) small (>0.2), moderate (>0.5), and or large (>0.8) (Cohen, 1992).

## 3 Results

**Figure 1:**
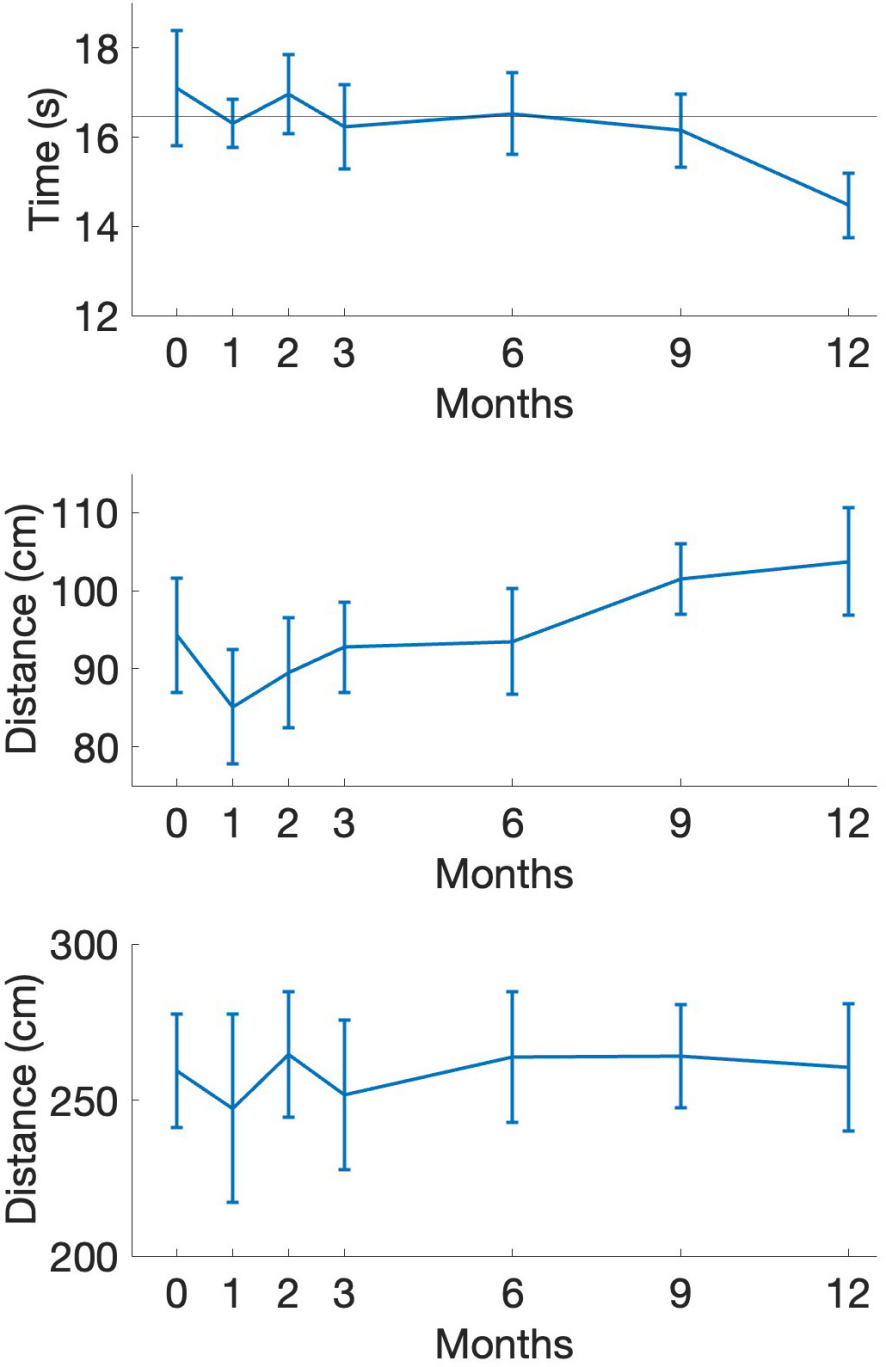
Functional measurements of FTSTS **(top)**, MDRT **(middle)**, and MSL **(bottom)** as a function of intervention time point. FTSTS, five times sit-to-stand; MDLT, Multidirectional Reach Test; MSL, Maximum Step Length Task. Horizontal line on FTSTS represents fall risk threshold (M = 16.47, see Section Discussion). Error bars represent 95% confidence interval.

**Figure 2:**
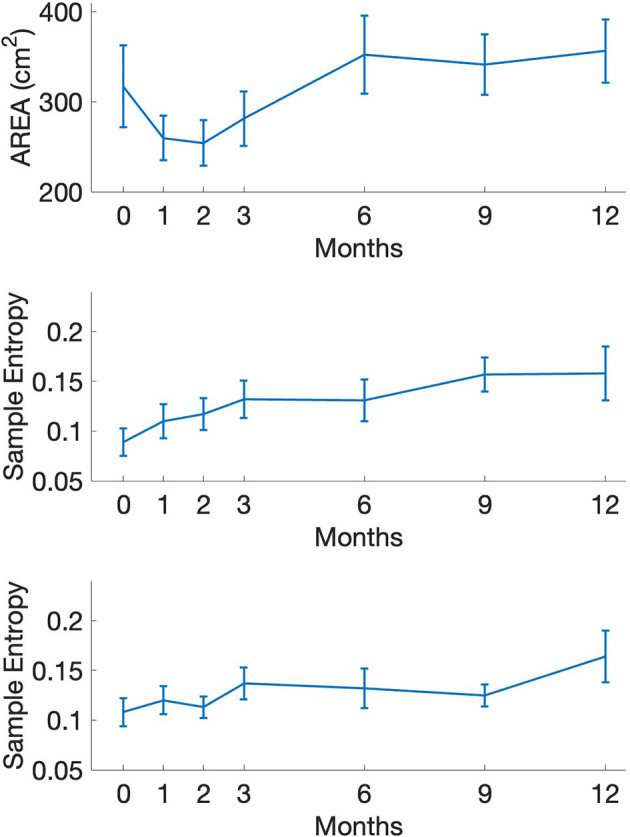
Postural sway indices of area **(top)**, sample entropy AP **(middle)**, and sample entropy ML **(bottom)** as a function of intervention time point. AP, anterior-posterior; ML, medial-lateral. Error bars represent 95% confidence interval.

**Figure 3:**
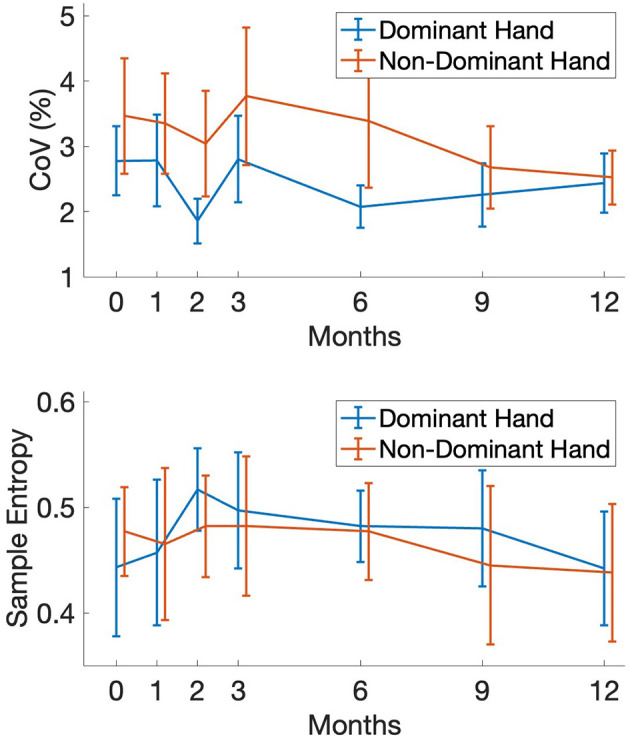
Force control of coefficient of variation **(top)** and sample entropy **(bottom)** for the dominant/non-dominant hands and as a function of intervention time point. Error bars represent 95% confidence interval.

Data presented in Table 1 highlights the overall effect size for the intervention time points following the initiation of the exercise intervention as compared to the averaged baseline metrics. A range of Cohen's *d* values were observed with a few intervention time points showing close to no correlation, while a majority of the other effect sizes were in the small to medium range. Gait speed changes at 1-month, 9-month, and 1-year intervention time points showed large effect size values. Trend patterns are shown in Figure 1 (functional metrics), Figure 2 (postural sway), and Figure 3 (force control) to illustrate the performance trajectories of the various neuromotor metrics when engaged in a sustained exercise program.

**Table 1:** Effect size values (*d*) for each intervention time point relative to baseline performance.

**Functional**	**BL-MO1**	**BL-MO2**	**BL-MO3**	**BL-MO6**	**BL-MO9**	**BL-1Y**
*FTSTS*	0.13	−0.11	0.03	−0.09	0.11	0.43
*MDRT*	0.351	−0.128	−0.259	−0.259	−0.713	−0.426
*MSLT*	−0.214	−0.25	−0.117	−0.261	−0.182	−0.211
*Gait Speed*	−0.801	−0.285	−0.596	−0.151	−0.901	−0.944
*ND Handgrip Strength*	0.552	0.453	0.392	0.092	0.148	0.389
*D Handgrip Strength*	0.513	0.329	0.858	0.376	0.298	−0.032
**Postural sway**	**BL-MO1**	**BL-MO2**	**BL-MO3**	**BL-MO6**	**BL-MO9**	**BL-1Y**
Area	0.131	0.253	0.010	0.056	0.150	0.208
SE-AP	−0.166	−0.218	−0.337	−0.328	−0.528	−0.538
SE-ML	−0.105	−0.040	−0.258	−0.216	−0.148	−0.501
**Force control**	**BL-MO1**	**BL-MO2**	**BL-MO3**	**BL-MO6**	**BL-MO9**	**BL-1Y**
ND CoV	0.078	0.558	−0.322	0.076	0.476	0.528
D CoV	0.011	1.078	−0.131	0.652	0.448	0.306
ND SE	0.079	−0.034	−0.034	0.00	0.214	0.259
D SE	−0.10	−0.552	−0.401	−0.289	−0.276	0.013

It is important to note that a negative d value indicates an increase from baseline, while a positive *d* value signifies a decrease from baseline. Effect size values were trivial (gray shade), small (light blue), moderate (medium-light blue), and large (medium blue). BL – baseline; MO – month; 1Y – 12-month timepoint; FTSTS – five times sit-to-stand tasks; MDRT – multidirectional reach task; MSLT – multidirectional step length task; ND – non-dominant; D – dominant; SE- sample entropy; AP – anterior-posterior; ML – medial-lateral, CoV – coefficient of variation.

Individuals showed faster completion times in the FTSTS tasks as well as an increased ability to maintain stability during the MDRT, indicating functional improvements with exercise. In terms of postural sway, initial assessments showed a restriction of movement that was altered during the later stages of the intervention where individuals exhibited increased sway magnitudes. Similarly, a positive increase in sample entropy was evident in both AP and ML directions of sway. A similar positive effect of the intervention can be seen in the COV value of force control with reductions occurring during the again in the later stages of the interventions.

## 4 Discussion

Possible benefits of exercise are evident for individuals with PD; however, significant work remains on the type of exercise, degree of physical activity, and targeted rehabilitation strategies required for attenuating disease progress. Here, we examined how a longitudinal multi-modal, non-contact boxing exercise program impacted the underlying mechanisms of neuromotor function in individuals with PD. This approach afforded a deeper understanding of PD impairments that can be used in the development of future rehabilitation guidelines. Overall, we found positive exercise-induced alterations within aspects of mobility, stability, and neuromotor force control following 12-month participation in this multi-modal boxing program. Such positive effects of exercise counter the typical 2–7% progressive decline of motor impairments in people with PD (Alves et al., 2005; Schrag et al., 2007).

Functional mobility metrics (indexed by reach and stepping tasks, gait speed, and FTSTS performance) index overall performance of a variety of daily living activities (putting dishes away, grabbing objects across the table) and can indicate the potential presence of a significant fall risk. Across these various measurements, different patterns of change occurred following engagement in the exercise intervention. First, no observable adaptation occurred for the multidirectional stepping task as most individuals performed on average similar to baseline indices (Figure 1). However, a consistent upward trend occurred for reaching scores from baseline throughout the 12-month intervention (Figure 1). Dynamic activities embedded within the boxing program likely contributed to the enhanced reach distances. The improved reach scores indicate an ability of the PD individuals to move their center-of-gravity toward a further stability boundary which has a direct impact on daily living activities. Additionally, these exercise activities, along with other circuit stations, potentially further promoted the increased gait speed shown by these individuals. Concomitantly, anecdotal accounts of improvements in general self-confidence likely aided in preserving the ability to perform functional activities while reducing fall risk and promoting independent living that should be examined in future studies (Yuan et al., 2020; Emig et al., 2021).

The FTSTS task has been shown to differentiate fall history within the PD population (Duncan et al., 2011). Understanding how exercise intervention impacts fall risk is vital. In terms of our multimodal exercise program, the results showed that at the 12-month assessment, five individuals performed the FTSTS task at or below a clinically defined fall risk threshold of 16 seconds (Duncan et al., 2011). As indicated in Figure 1 (top), all of our PD individuals were above this cut-off mark (M = 16.47) at baseline and at risk of experiencing a fall. While it is not possible to link the specific exercise activities that contributed to this positive change, the finding is in agreement with the notion that a general, multi-modal exercise program promotes enhanced functional mobility (Bouça-Machado et al., 2020) that reduces fall risk in the PD population. Gaining further understanding of the widespread impact of boxing interventions appears worthwhile given the current findings and the potential positive impressions on quality independent living needs.

An interesting and meaningful finding was observed when comparing the postural control changes relative to sway magnitude and structure (Figure 2). Engagement in the boxing program resulted in individuals displaying increased movement (ie., large sway area) during static standing conditions while also producing postural sway that reflected increased irregularity. In contrast to typical interpretations that highlight reduced amounts of sway as an indicator of better postural control, our PD population showed increased sway magnitudes. Given the heterogeneous PD diagnoses and lack of previous exercise involvement, it appears that our individuals initially used rigid, protective balance strategies that minimized motion and disturbances to maintain postural stability. At baseline levels, the lower complexity indices (i.e., sample entropy) of postural sway further support the use of such a strategy (Duarte and Sternad, 2008; Manor et al., 2010), which also is a neuromotor trait that is reflective of the classic “Loss of Complexity” trait of aging and disorders (Lipsitz, 1992).

With exercise engagement, increased amounts of sway can be viewed as a positive adaptation facilitating enhanced postural stability and, when coupled with the complexity, results demonstrate robust, positive changes in balance performance. Specifically, this optimal movement variability perspective (Stergiou et al., 2006) highlights increased physiological signal irregularity that offers increased adaptability. In our findings, exercise involvement resulted in increased irregularity throughout the entire intervention period for most PD individuals. Such changes in postural sway complexity reflect a collective reorganization of the COP trajectories that indicates an adaptability trait. This higher degree of complexity allows for individuals to quickly adjust control strategies to internal perturbations. Overall, the observed shifts in postural sway complexity are more reflective of a healthy neuromuscular system and counteract the known disease progress timeline.

Isometric force production has been shown to be diminished for PwPD for tasks of the upper and lower limbs (Corcos et al., 1996; Hammond et al., 2017; Chung et al., 2023). However, there is a significant absence of data on longitudinal changes in force control for PwPD with or without exercise intervention. In light of this, our study provides important resource contributions in this arena given its duration. Examining changes in isometric force control within our cohort, it appears that our linear and non-linear assessments demonstrate unique patterns of change across the year of training. Generally, over the year, CoV improved, most notably for the non-dominant hand, while SampEn values were generally stable with minimal change. Taken together, these findings indicate that linear and non-linear measures of isometric force control in our cohort of PwPD did not degrade over the year-long study period. Moreover, the improvements in force steadiness (CoV) should be considered a positive effect given the natural declines associated with PD progression. Therefore, evidence of attenuated loss appears to support the robust neuroplasticity associated with exercise interventions for individuals with a PD diagnosis (Corcos et al., 2013). The physiological contributions to diminished force control with aging are attributable to motor unit remodeling, loss, and diminished muscle fiber twitch kinetics (Piasecki et al., 2016). The deficits in dopaminergic circuits within motor networks, distributed across higher cortical centers to the brain stem, further exacerbate age-related force control loss (Corcos et al., 1996; Fellows and Noth, 2004; Chung et al., 2023). There is an urgent need for large-scale trials to identify longitudinal assessments of force control changes in PwPD with and without exercise intervention.

### 4.1 Limitations

While the male population represents the majority of PD diagnosis, there remains a need to understand how exercise engagement impacts all individuals with PD. Thus, expanding the current findings to wider demographics beyond the six males used for this case study example is a critical next step. Also, the lack of a time-matched control group limits our ability to discern between inherent neuroplastic improvements and practice effects supporting motor learning changes alone. A small number of investigations have explored long-term adaptations to exercise within the PD population and continued efforts are needed to target disease attenuating effects through engagement of physical exercise programs. As partially evident in our data, the heterogeneity of PD progressions likely influences the effectiveness of an exercise intervention to improve PD symptomology, and future studies will need to consider personalized, symptom-specific exercise plans.

## 5 Conclusion

Longitudinal involvement in a multi-modal, non-contact boxing exercise program had a profound effect on neuromotor components of mobility, stability, and force control in a small cohort of PwPD. While an effect for some clinical metrics is lacking, it is important to note that the pattern of results did not show the typical decline of PD over a one-year period. The novel findings related to the neuromotor function of PwPD provide insights into exercise responses and the positive impact of long-term physical activity participation. Lastly, the evidence contributes to the growing literature of the positive benefits with multi-modal exercise programs, including non-contact boxing, and warrants further investigations using larger cohorts and control comparisons.

## Data Availability

The raw data supporting the conclusions of this article will be made available by the authors, without undue reservation.
